# Study protocol: randomized controlled trial of opioid-free vs. traditional perioperative analgesia in elective orthopedic surgery

**DOI:** 10.1186/s12891-021-03972-9

**Published:** 2021-01-23

**Authors:** Elaine Z. Shing, Daniel Leas, Caleb Michalek, Meghan K. Wally, Nady Hamid

**Affiliations:** 1 Carolinas Medical Center, Atrium Health Musculoskeletal Institute, P.O. Box 32861, Charlotte, NC 28232 USA; 2 Carolina Neurosurgery and Spine Associates, Charlotte, NC USA; 3 OrthoCarolina Research Institute, Charlotte, NC USA; 4 OrthoCarolina Shoulder and Elbow Center, Charlotte, NC USA

**Keywords:** Pain management, Opioid crisis, Elective surgery

## Abstract

**Background:**

The medical community is beginning to recognize the contribution of prescription opioids in the growing national opioid crisis. Many studies have compared the safety and efficacy of alternative analgesics to opioids, but none utilizing a completely opioid-free perioperative protocol in orthopedics.

**Methods:**

We developed and tested an opioid-free perioperative analgesic pathway (from preoperative to postoperative period) among patients undergoing common elective orthopedic procedures. Patients will be randomized to receive either traditional opioid-including or completely opioid-free perioperative medications. This study is being conducted across multiple orthopedic subspecialties in patients undergoing the following common elective orthopedic procedures: single-level or two-level ACDF/ACDA, 1st CMC arthroplasty, Hallux Valgus/Rigidus corrections, diagnostic knee arthroscopies, total hip arthroplasty (THA), and total shoulder arthroplasty/reverse total shoulder arthroplasty (TSA/RTSA). The primary outcome measure is pain score at 24 h postoperatively. Secondary outcome measures include pain scores at additional time points, medication side effects, and several patient-reported variables such as patient satisfaction, quality of life, and functional status.

**Discussion:**

We describe the methods for a feasibility randomized controlled trial comparing opioid-free perioperative analgesics to traditional opioid-including protocols. We present this study so that it may be replicated and incorporated into future studies at other institutions, as well as disseminated to additional orthopedic and/or non-orthopedic surgical procedures. The ultimate goal of presenting this protocol is to aid recent efforts in reducing the impact of prescription opioids on the national opioid crisis.

**Trial registration:**

The protocol was approved by the local institutional review board and registered with clinicaltrials.gov (Identifier: NCT04176783) on November 25, 2019, retrospectively registered

## Background and rationale

Opioids have long been used in various forms for pain control in the medical field. While there is demonstrated analgesic effect of these compounds [1], they are also associated with a number of side effects, including constipation, nausea/vomiting, hyperalgesia [2, 3], delirium [4], opioid dependence/withdrawal, and even respiratory depression/death [5]. Their use for acute pain management has undergone a logarithmic increase in the past twenty years, which has also brought a concomitant rise in opioid-induced side effects. Patient expectations of opioid medications has driven a rapid rise in outpatient opioid prescriptions for both short and long-acting opioids [6], which have additionally shown substantial addiction potential. It has been found that two-thirds of patients taking opioids 3 months after elective surgery are still on opioids at an average of 4.8 years later [6].

The existing literature on opioid prescribing for musculoskeletal conditions has consistently demonstrated frequent over-prescribing, leading to unused pills available for nonmedical use or diversion [7–12]. These prescriptions have become a source of significant mortality in the United States, with nearly 48,000 opioid-related overdose deaths in 2018 alone (illicit and prescription combined) [13]. However, the rate of prescription-opioid deaths encouragingly dropped for the first time in nearly 10 years, decreasing from 17,000 to 15,000 deaths between 2017 and 2018 [13].

This decrease in prescription-opioid death rates can largely be attributed to widespread efforts to reduce provider reliance on opioids. The scientific community now has an improved understanding of the risks and benefits of various forms of pain medication. Efforts have been made to identify synergistic compounds to use for acute pain management in the perioperative time period, all of which report some degree of opioid-sparing effects. These studies have focused on the safety and efficacy of gabapentinoids [14–16], local administration of sodium-channel blockers such as lidocaine and bupivacaine [17–19], opioid-free anesthesia [20–22], cryotherapy [23, 24], dexamethasone [25, 26], and non-steroidal anti-inflammatories (NSAIDs) and acetaminophen [27–33].

The most robust adjuvant effects have been reported with NSAIDs and acetaminophen. Some studies indicate that there may be a synergistic effect between these two to reduce postoperative opioid intake [34]: oxycodone 15 mg and ibuprofen 600 mg have comparable treatment efficacy as measured by number needed to treat (NNT) (2.3 vs. 2.4, respectively). Ibuprofen 800 mg has the highest treatment efficacy (NNT 1.6) of many reported analgesic combinations [35].

At this time, no study has examined the possibility of utilizing a multi-modal perioperative analgesic pathway that does not include some form of opioid medication in orthopedic patients. We report an ongoing feasibility randomized controlled trial (RCT) designed with the objective to investigate the safety and efficacy of opioid-free pain management in elective orthopedic procedures and determine if opioid-based analgesia is needed in orthopedic surgery. This is, to our knowledge, the first investigation of its kind. Patients in the opioid-free analgesia arm did not receive any opioid medications in the pre-, intra-, and postoperative phases through the time they followed up in clinic (including the general and local anesthesia protocols). This article describes our opioid-free clinical pathway in detail with review of the existing literature to justify each component. The ultimate goal is that this protocol may be replicated for future studies and potentially incorporated into a perioperative clinical pathway. Should this study demonstrate equivalent efficacy of opioid-free and opioid-based analgesic protocols, then its findings may also help reduce the amount of prescribed opioid medications for patients undergoing elective orthopedic surgery.

## Methods: trial design, patient selection, and intervention

### Overview

**Fig. 1:**
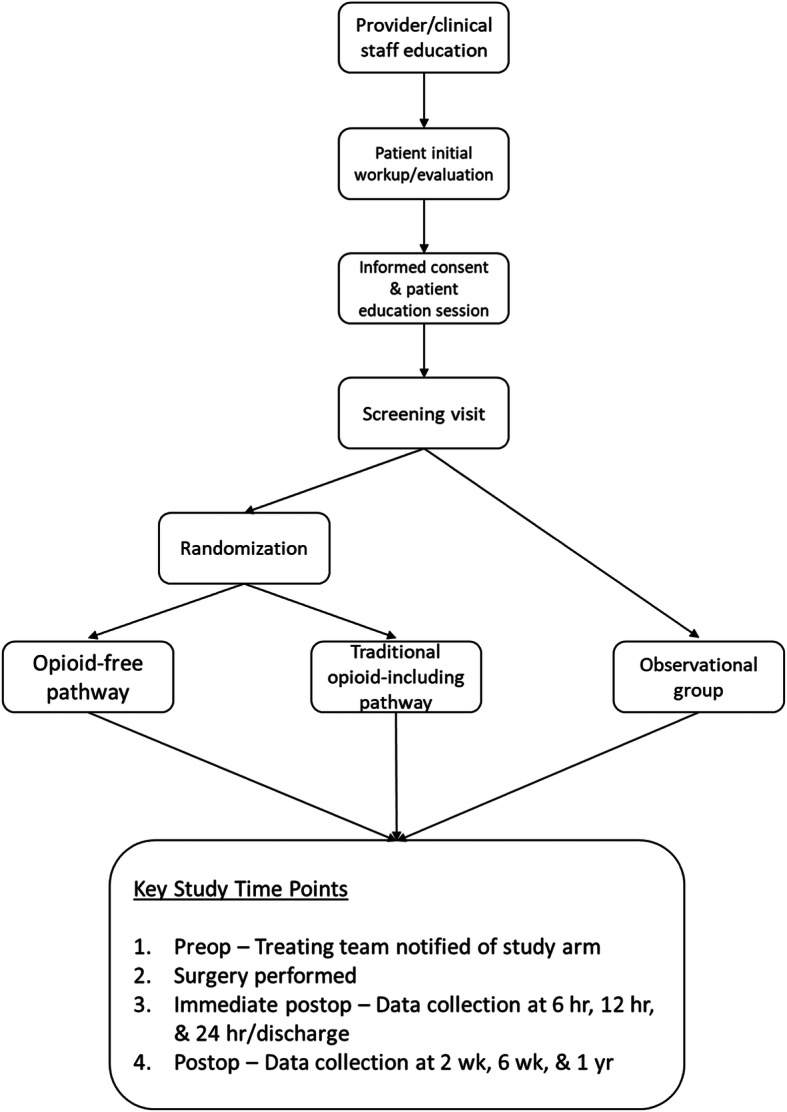
Flow diagram illustrating study protocol

This parallel group RCT was performed at a single institution, which is a large private orthopedic practice with an affiliated research institute, across 6 subspecialties (Shoulder & Elbow, Hip & Knee, Hand, Sports, Spine, Foot & Ankle). Specifically, surgeons from this one practice enrolled their patients; patients had surgery across six facilities that were either hospital-affiliated or private ambulatory surgery centers (Atrium Health Mercy, Charlotte Orthopedic Hospital, Carolinas Medical Center One-Day Surgery, Charlotte Surgery Center, Mallard Creek Surgery Center, and Matthews Surgery Center). The study also included an observational arm for those patients who did not wish to be randomized. The protocol was approved by the local institutional review board and registered with clinicaltrials.gov (Identifier: NCT04176783; Protocol version 7; October 11, 2019), retrospectively registered (URL: https://clinicaltrials.gov/ct2/show/NCT04176783?term=NCT04176783&draw=2&rank=1). Figure 1 provides a summary of the clinical pathways.

A draft medication protocol was developed for each subspecialty, covering preoperative, intraoperative, post-anesthesia care unit (PACU), postoperative, and discharge settings based on the literature. A steering committee consisting of physicians, anesthesiologists, nurses, and the research team was created to develop these protocols. Specifically, one orthopaedic surgeon was included from each of the following subspecialties: Shoulder & Elbow, Spine, Foot & Ankle, Hand, Sports, and Hip & Knee. One orthopaedic surgery resident was included. Finally, four anesthesiologists to represent both hospital systems were on the steering committee. These steering committee members are experts in their respective fields and utilized existing literature (summarized in the background section) to inform the inclusion of various non-opioid analgesics in the protocols. Pilot testing was performed on a group of shoulder arthroplasty patients (data published previously in a case series [36]). The final protocols are presented in Table 2. While the choice of each medication in these protocols was informed by the literature [14–35, 37] and expert consensus of the steering committee, the proposed study will assess the efficacy of these protocols for pain control in a larger group of elective orthopedic surgery patients.

Patients were randomized to one of two arms. The treatment group included treatment without the use of opioids. The control group included a traditional opioid-based pain protocol, agreed upon by the treating surgeons. The observational group consisted of patients who were eligible for the study but were not willing to be randomized to a postoperative pain management pathway. They were treated based on patient/surgeon preference and included in an associated cohort study.

The study population consisted of patients aged > 18 undergoing the following routine elective orthopedic surgical procedures: single-level or two-level Anterior cervical discectomy and fusion/Anterior cervical disc arthroplasty (ACDF/ACDA), 1st carpometacarpal (CMC) arthroplasty, Hallux Valgus/Rigidus corrections, diagnostic knee arthroscopies, total hip arthroplasty (THA), and total shoulder arthroplasty/reverse total shoulder arthroplasty (TSA/RTSA). Eligible patients who provided informed consent were randomized to one of the two study arms. Outcomes were assessed at multiple time points – 6, 12, and 24 h postoperatively, then 2 weeks, 6 weeks, and 1 year postoperatively.

### Care team education

A key element of the clinical protocol was providing physicians and clinical/research staff with education on opioid medications and their role in perioperative pain-relief. Studies have identified knowledge gaps in providers managing chronic [38] and acute postoperative pain [39]. However, provider-directed education on opioid-related issues can be successful in decreasing postoperative opioid use [40–42]. As such, institutions are beginning to recommend interventions for providers regarding these subjects [22]. Our study employed multiple in-service sessions for investigators, research staff, and nurses/hospital support staff to educate and prepare them for the opioid-free clinical pathway. These sessions outlined the purpose of the study, highlighted the dangers and pitfalls of opioid use, and provided evidence for pharmacologic alternatives. We recognized that this pathway represented a significant change to the typical work-flow for many care team members including anesthesiologists, nurses, and surgeons. We anticipated skepticism among some health care workers and found that pre-study meetings and in-service training sessions helped to offer clarity about the specifics of the clinical pathway and instilled confidence that patients’ comfort levels would remain a high priority.

### Patient selection, recruitment, and randomization

#### Eligibility and recruitment

Eligibility was determined based on the inclusion and exclusion criteria listed in Table 1. Eligible participants were identified after initial evaluation by a staff physician, determined to require one of the qualifying procedures, and approached for informed consent by the physician and research staff. At this time, patients watched an introductory video explaining the purpose of the clinical trial. The physician also led an in-depth preoperative discussion explaining each approach to analgesia (opioid vs. opioid-free) and postoperative expectations. The inclusion of this process was driven by recent data showing decreased use of postoperative opioid medications after patients received preoperative opioid education [43–46]. If patients declined to participate in the randomized trial, they were presented with the option of enrolling in the observational group where they would be able to select either the control opioid-containing arm or the opioid-free arm. There were no restrictions on patient care due to enrollment in the study.

**Table 1:** Eligibility Criteria

**Inclusion Criteria:** • Patient is scheduled to undergo one of the following procedures: • Primary single-level or two-level ACDF or ACDA for degenerative disease • Primary 1st CMC arthroplasty • Primary Hallux Valgus or Hallux Rigidus correction • Diagnostic knee arthroscopy +/− meniscal debridement • Elective primary total shoulder or reverse total shoulder arthroplasty • Primary total hip arthroplasty	**Exclusion Criteria:** • Revision surgery for one of the study-specific procedures • Chronic opioid therapy – per investigator discretion • Significant liver disease – (NOTE: Patients with a history of liver disease had a hepatic panel drawn that was reviewed by the study investigator to assess if the values were within acceptable limits for inclusion in the study) • Fracture or soft tissue injury • Sickle cell disease • Workers compensation • Alcohol dependence • Contra-indication to regional anesthesia • History of gastrointestinal (GI) bleeding or peptic ulcer • History of bleeding problems • Patients taking anticoagulants, not including aspirin (only applied to Randomized portion of study. These patients could still participate in Observational Control Group) • Renal insufficiency – Creatinine clearance less than 30 mL/min (only applied to patients having surgery requiring NSAID treatment) • Hammertoe in isolation (Hallux Valgus/Rigidus exclusion only) • Concomitant meniscal repair or microfracture (Knee Arthroscopy exclusion only) • Ineligible for spinal anesthesia (THA exclusion only) • Previous ipsilateral hip surgery, not including hip scope (THA exclusion only) • Allergy to non-steroidal anti-inflammatory medications (NSAIDs)

#### Screening visit

Participants underwent an initial screening visit that included a series of questionnaires (dependent upon the type of surgery being performed) and physical exam. The questionnaires, described in the outcomes section below, asked about level of pain, function, and satisfaction with pain control. The exam included an assessment of range of motion, when applicable. All patients were asked to complete a Resilience Questionnaire (Resilience Scale 5, [47]) at this visit.

All patients underwent a blood draw (5 ml) to confirm eligibility based on creatinine clearance (CrCl), which was calculated with the Cockcroft-Gault Formula. Patients with a CrCl < 30 mL/min were excluded from the study. Patients with CrCl between 30 and 60 mL/min received a half-dose of NSAIDs if enrolled in the intervention arm of the study. Patients with reported liver disease received a second blood draw to evaluate hepatic liver enzymes and ensure safe dosing of acetaminophen.

#### Randomization

Patients were randomized in a 1:1 fashion to one of the treatment groups – Control (with opioids) or Intervention (opioid-free). Randomization occurred within 1 week prior to surgery and was performed with a random number generator in REDCap by research staff [48]. This random number generator was built into the REDCap system such that the study coordinators and investigators were unaware of what the randomization schedule would be. The allocation was then communicated to the surgical team via email. A randomization form was placed on the patient chart prior to surgery. Patients were not blinded to the treatment arm.

Once randomized to an arm, the treating team of surgeon, anesthesiologist, and clinical staff were notified of the result. There was no blinding involved in this study. Patients were identified as being a part of the study, and the corresponding treatment arm was listed in their hospital chart. Reminders of each patient’s treatment designation were provided to the anesthesiology team in the preoperative holding area on the day of surgery.

#### Sample size calculation

Estimated sample size was calculated using data from a previous study comparing the pain numeric rating scale (NRS) at 24 h between patients receiving opioid medications or not [36]. This was a noninferiority design with a 2-point margin. The mean pain score for the traditional group was 3.2 and the mean pain score for the non-opioid group was 2.5 with a pooled standard deviation of 2.5. An alpha level of 0.5 and a minimum power of 80% yielded 50 patients per group per procedure. Therefore, the target sample size was 300 completed patients at 24 h in the traditional group and 300 completed patients at 24 h in the opioid-free group.

### Intervention

Patients in the opioid-free treatment arm did not receive any opioid medications from the preoperative period to the postoperative period. The control arm included traditional opioid-based analgesia. Medications in the treatment arm were selected based on findings from current literature on opioid-alternatives. Medications in the control arm were included as usual perioperative analgesic options. Some variations to the treatment pathways existed by procedure type. Tables 2 and 3 illustrate the full protocol of analgesic administration for each arm. As described above, the steering committee developed the protocols based on peer-reviewed literature and expert consensus among the committee.

**Table 2:** Opioid-free analgesic protocol for each subspecialty and perioperative phase

		Spine (ACDF/ACDA)	Foot&Ankle (HalluxValgus/Rigidus)	Hip (THA)	Knee (Knee Scope)	Shoulder (TSA/RTSA)	Hand (1st CMCarthroplasty)
**PreOp**	Gabapentin (PO)	300 mg (or doublecurrent up to 900)	300 mg	300 mg	300 mg	300 mg	300 mg
	Tylenol (IV)	1000 mg (maysubstitute to PO ifIV Tylenol is notavailable)	1000 mg (maysubstitute to PO if IVTylenol is notavailable)	1000 mg (maysubstitute toPO if IV Tylenolis not available)	1000 mg (maysubstitute to PO if IVTylenol is not available)	1000 mg (maysubstitute to PO if IVTylenol is not available)	1000 mg (maysubstitute to POif IV Tylenol isnot available)
	Meloxicam (PO)	15 mg	15 mg	15 mg	15 mg	15 mg	15 mg
	Scopalamine Patch	Yes (if age < 75)	Yes (if age < 75)	Yes (if age < 75)	Yes (if age < 75)	Yes (if age < 75)	Yes (if age < 75)
	Single-shot Spinal			1.5-2.0 cc 0.75%Bupivacaine(upright in OR)			
**IntraOp**	Local Injection	0.5% Marcaineplain 5 cc	None	CERT	0.25% Marcaine plain20-30 mL (based onbody habitus)	Exparel 20 cc(suspended in 20 cc0.25% Marcaine) **or**CERT	None
	Block		Regional Ankle Block			Brachial Plexus Block	Brachial PlexusBlock
	Toradol	30 mg IV push ×1 dose (adjust forCrCl) - (given atend of sx or inPACU)	30 mg IV push × 1dose (adjust for CrCl)- (given at end of sxor in PACU)	30 mg IV push × 1 dose (adjustfor CrCl) - (givenat end of sx orin PACU)	30 mg IV push × 1 dose (adjust for CrCl)- (given at end of sx orin PACU)	30 mg IV push × 1dose (adjust for CrCl) -(given at end of sx orin PACU)	30 mg IV push × 1 dose (adjustfor CrCl) - (givenat end of sx orin PACU)
	Zofran (IV)	4 mg	4 mg	4 mg	4 mg	4 mg	4 mg
	Decadron	10 mg	10 mg	10 mg	10 mg	10 mg	10 mg
**PACU**	Cryotherapy		Yes	Yes	Yes	Yes (q8h)	Yes
	Tylenol (IV) - *up todaily maximumdose allowable perinstitution	1000 mg q6h PRN*	1000 mg q6h PRN*	1000 mg q6hr ×4doses (continuesinto postop) (maysubstitute to PO ifIV Tylenol is notavailable)	1000 mg q6h PRN*	1000 mg q6h PRN*	1000 mg q6h PRN*
	Tylenol (PO) - *upto daily maximumdose allowable perinstitution	500 mg q6h PRN*	500 mg q6h PRN*		500 mg q6h PRN*	500 mg q6h PRN*	500 mg q6h PRN*
**PostOp**	Cryotherapy		optional	Yes (q8h)	optional	Yes (q8h)	optional
	Toradol (IV)	15 mg q8h ×4doses (can beredosed to atotal of 30 mg q8h)		15 mg q8h ×5doses (can beredosed to a totalof 30 mg q8h) -available for rescue		15 mg q8h ×4 doses(can be redosed to atotal of 30 mg q8h)	
	Tylenol (IV) - *up todaily maximumdose allowableper institution	1000 mg q6h PRN*		1000 mg q6h PRN*		1000 mg q6hr PRN*	
	Tylenol (PO) - *upto daily maximumdose allowable perinstitution	500 mg q6h PRN*		500 mg q6hr PRN*		500 mg q6hr PRN*	
	Gabapentin	300 mg PO(or double currentup to 900)		300 mg q8h		300 mg q8h	
	Meloxicam			7.5 mg q24h			
	Decadron			10 mg (1 dose -postop day #1)			
**Discharge**	Cryotherapy		Yes		Yes	Yes	Yes
	Toradol (PO)		10 mg TID x 5d		10 mg TID x 5d		10 mg TID x 5d
	Meloxicam	15 mg Q24hr × 14d	(After 5 days ofToradol) 15 mg q24hrx 28d	15 mg q24h x 28d	(After 5 days ofToradol) 15 mgq24hr x 28d	15 mg q24hr x 28d	(After 5 days ofToradol) 15 mgq24hr x 28d
	Gabapentin	300 mg PO(or double currentup to 900) ×14 day(initiate wean onpatients with preopuse)	300 mg q8h × 14d	300 mg q8h × 14d	300 mg q8h × 14d	300 mg q8h × 14d	300 mg q8h × 14d
	Tylenol	500 mg q4h PRN	500 mg q4h PRN	325 mg q4h PRN	500 mg q4h PRN	500 mg q4h PRN	500 mg q4h PRN
	ASA			81 mg BID x 28d		81 mg BID x 28d	

* indicates dosing up to daily maximum allowed

**Table 3:** Traditional opioid-including analgesic protocol for each subspecialty and perioperative phase

		Spine (ACDF/ACDA)	Foot&Ankle (HalluxValgus/Rigidus)	Hip (THA)	Knee (Knee Scope)	Shoulder(TSA/RTSA)	Hand (1st CMCarthroplasty)
**PreOp**	Gabapentin (PO)			300 mg			
	Tylenol (IV)			1000 mg (may substituteto PO if IV Tylenol is notavailable)			
	Meloxicam (PO)			15 mg			
	Scopalamine Patch			Yes (if < 75 y.o.)			
	Single-shot spinal			1.5-2.0 cc 0.75%Bupivacaine(upright in OR)			
**IntraOp**	Local injection			CERT	0.25% Marcaine plain5-10 mL (range basedon body habitus)		
	Block		Regional Ankle Block			Brachial Plexus Block	Brachial Plexus Block
	Toradol			30 mg IV push x 1 dose(adjust for CrCl) *(given atend of sx or in PACU)*			
	Zofran (IV)	4 mg	4 mg	4 mg	4 mg	4 mg	4 mg
	Decadron	10 mg		10 mg			10 mg
**PACU**	Cryotherapy			Yes			
	Tylenol (IV) - *up to dailymaximum dose allowableper institution			1000 mg q6h ×4 doses(continues into postop)(may substitute to PO ifIV Tylenol is not available)			
**PostOp**	Dilaudid (IV)	0.5-1 mg q3h PRN		0.5-1 mg q3h PRN		0.5-1 mg q3h PRN	
	Norco	5/325 mg 1-2q4h PRN				5/325 mg 1-2q4h PRN	
	Tramadol	50 mg q6h PRN					
	Cryotherapy		optional	Yes (q8h)	optional	optional	optional
	Toradol (IV)			15 mg q8h ×5 doses(can be redosed to atotal of 30 mg q8h) -available for rescue			
	Decadron (IV)			10 mg IV push x 1 dose			
	Tylenol (IV) - *up to dailymaximum dose allowableper institution			1000 mg q6h PRN*			
	Tylenol (PO) - *up to dailymaximum dose allowableper institution			500 mg q6hr PRN*			
	Gabapentin			300 mg q8hr			
	Meloxicam			7.5 mg q24hr			
	Oxycodone			5-10 mg q4h PRN			
**Discharge**	Norco	5/325 1-2 q4h PRN(30 tab)	Norco 5/325 1-2tabq4h PRN (40 tab)				5/325 1-2tab q4h PRN(30 tab)
	Percocet			5/325 mg 1-2q4h PRN(#80)	5/325 1 tab q4hPRN (30 tab)	5/325 mg 1-2q4hPRN (#80)	
	ASA			81 mg BID x 28d		81 mg BID x 28d	
	Meloxicam			15 mg q24h x 28d			
	Gabapentin			300 mg q8h x 14d			

* indicates dosing up to daily maximum allowed

We employed several strategies to improve adherence to study protocols. First, we took measures to ensure that medical staff were clearly aware of which study arm each patient was enrolled in. The treating surgeon and key staff contacts at the surgery location’s preoperative, anesthesia, post anesthesia care, and postoperative units received an email notification with the patient’s assigned study arm at the time of randomization (1 week prior to surgery) and 2 days prior to the surgery. This email also contained a reminder of the medication protocol specific to that case. A notification sheet and copy of the medication protocol was placed on the front of the patients’ hospital charts along with an opioid-free wristband if the patient was randomized into the opioid-free arm of the study. Secondly, all patients received a diary after their surgery to record medication usage in the first 2 weeks of the postoperative period. This diary helped monitor protocol adherence, at least in self-reported form.

Importantly, patients and care team members were also free to communicate with the treating physician any levels of discomfort found to be unsatisfactory to the patient. If a patient had exhausted all options in the opioid-free pathway, they were given the option to receive opioids. Upon discharge, patients were given instructions to contact their physician for unacceptable levels of pain to receive an outpatient prescription of an opioid. However, no “rescue” or “just in case” prescriptions were given to patients upon discharge to avoid prescription diversion and unnecessary home stores of opioids. Additionally, if a patient deviated from the medication protocol assigned to them, they were not considered study failures. Instead, we continued to follow the patient through study completion and continued to track patient-reported outcomes and total opioid consumption.

## Methods: data collection and outcome measures

### Baseline assessments and frequency of follow-up assessments

A full list of assessments obtained at each time point is summarized in Table 4.

**Table 4:** Full list of assessments obtained at each time point

Procedure	Preop/Screen	Op	6 h	12 h	24 h	2 week	6 week	1 year
ICF	X							
Demographic	X							
Comorbidities	X							
Resilience Questionnaire (RS-5)	X							
Central Sensitization Inventory	X							
Creatinine Clearance	X							
Randomization	X *(within one week prior to surgery)*							
Complications (nausea, constipation, falls)			X^a^	X^a^	X^a^	X	X	X
Delirium Score (CAM25)^a^			X^a^	X^a^	X^a^			
Patient Comfort Level (NRS)^a^			X^a^	X^a^	X^a^			
ConMeds (specifically anti-emetic/nausea and pain medication)	X	X	X^a^	X^a^	X^a^	X	X	X
Surgical & Hospital-Stay Information		X						
Pain Score (NRS), current	X		X	X	X	X	X	X
Pain Score (NRS), average	X					X	X	X
Constipation Questionnaire (PAC-SYM)	X					X	X	X
Patient Reported Outcomes Questionnaires *(specific to applicable surgery)*	X					X	X	X
Patient Pain (NRS) Diary±						X		
Patient Comfort (NRS) Diary±						X		
Patient Pain Medication Diary±						X		
Patient Collar Compliance Diary± *(ACDF/ACDA patients only)*						X		

^a^ Only applies to inpatient procedures

± The diaries will be completed daily from day of discharge until the 2 week visit. They will be collected at the 2 week visit

We obtained baseline assessments during each patient’s screening visit. These measures included, but were not limited to, demographics, comorbidities, subjective pain ratings, constipation ratings, patient reported outcomes specific to the planned procedure, and resilience ratings.

Initial follow-up assessments were obtained at 6, 12, and 24 h after surgery. Patients were asked to return for postoperative follow-up visits at 2 weeks, 6 weeks, and 1 year after surgery. These visits were scheduled to be at routine visits that were already a part of our investigators’ standard-of-care treatment time tables. Patients that were unable to attend any in-person visit were asked to completed all study patient-reported outcomes via phone call or REDCap electronic survey for that visit to facilitate retention and optimal follow-up. Pain level, medication usage, constipation level, complications, and patient reported outcomes were gathered during these visits.

Each patient received a diary upon hospital discharge to record their pain levels, overall comfort/satisfaction levels, and medication usage. Patients who underwent ACDF/ACDA surgery were also asked to record their compliance with soft collar equipment. Information from the diaries was collected at the 2-week postoperative visit.

### Primary outcome measures

The primary outcome measure was pain at 24-h postoperatively. If the patient was in-hospital, the pain rating was obtained by the nurse. If the patient had been discharged, they were called by research staff to obtain a pain rating. This outcome was measured on a 0-10 NRS, a reliable and valid measure of present-moment subjective pain [49].

### Secondary outcome measures

Secondary outcomes of interest included pain ratings (NRS) at the additional time points noted previously as well as other clinical and patient-reported variables.

Basic clinical information was collected at time of surgery or hospitalization, including length of hospital stay, intraoperative complications, and length of surgery. Morphine milli-equivalents were recorded in-hospital, and post-operative opioid use was recorded after discharge. Medication side effects were recorded as episodes of delirium, number of falls, and patient reported nausea/constipation.

Several patient-reported variables were collected throughout the duration of the study and included nausea/constipation, satisfaction with pain control and surgical experience, quality of life, resilience, and functional status specific to the surgery performed. Functional outcomes were assessed with validated, standardized questionnaires (e.g. American Shoulder and Elbow Surgeons Shoulder Score for TSA/RTSA patients [50] and Foot and Ankle Ability Measure for hallux valgus/rigidus patients [51]). We also collected qualitative data from patients in the control group regarding unused opioid pills. They were asked to describe if and how the pills were disposed of or secured away if not disposed.

### Covariates and confounders

Several variables were measured as potential covariates, including pre-operative opioid use, pre-operative pain scores, concomitant procedures, medical comorbidities (based on the Charlson Comorbidity Index), BMI, alcohol or other non-prescribed medication use to help with comfort, tobacco use, and tourniquet time (when applicable). Basic demographic information was also noted.

## Methods: data management, analysis, & additional information

### Data management

Data were collected by research staff and entered into REDCap (http://project-redcap.org/) [46] on at least a weekly basis. REDCap is a secure web application designed to support data capture for research studies. The program provides audit trails for tracking data manipulation and user activity, as well as automated export procedures for data downloads to common statistical packages (Excel, SPSS, SAS, Stata).

Study information is de-identified by removing PHI and using coded subject identifiers. Study data is kept in locked cabinets/rooms only accessible by research staff. Electronic data is kept in an access-privileged, password-protected, encrypted database.

Adverse events were collected as part of the surgical and follow-up source documents. They were collected via patient reporting, questionnaires, and chart review. Serious Adverse Events that were “related,” “probably related” or had an “unknown” relatedness to the study procedure were reported via secure email to the data safety monitoring board as they occurred. These occurrences were reviewed every other month or as needed by the data safety monitoring board, which is made up of members of the OrthoCarolina Research Institute (OCRI) Research Advisory Committee. At each meeting, the study was reviewed for adverse events, serious adverse events, and overall feasibility issues. Additionally, the board conducted internal audits for this clinical trial. Initial auditing occurred after surgery and data collection had been completed for one patient in each study group per subspecialty. After that point, auditing has occurred on an as-needed basis with an internal audit happening at least once per year.

### Statistical analysis

The primary outcome of pain at 24-h postoperatively will be assessed for normality using visualization methods, including QQ plots as well as statistical analysis tests including Shapiro-Wilk. If these data are normally distributed, an independent t-test will be used to compare pain at 24-h postoperatively. If these data are non-normal, a Mann-Whitney U test will be used to compare pain at 24-h postoperatively between treatment groups.

Normality testing as previously described will be evaluated for all continuous secondary outcomes including pain at additional postoperative time points, length of stay, morphine milliequivalents, satisfaction, and scores from patient reported outcome measures. The appropriate statistical test, either independent t-tests or Mann-Whitney U tests, will be used to compare these outcomes between treatment groups. Categorical outcomes such as intraoperative complications, medication side-effects, and other postoperative complications, will be compared between treatment groups using a Chi-Square of Fishers Exact test.

Statistical associations between covariates and outcomes will be evaluated using the appropriate statistical methodology as previously described. Multivariable linear regression models will be appropriately fitted to continuous data based on the distributions of that data. Multiple logistic regression models will be fitted for all dichotomous outcome variables. Appropriate variable selection methods and model fit statistics will be used to determine the best fitting model to determine the effects of the treatment after adjusting for significant effects of covariates.

Finally, a per protocol analysis is planned. There is no plan to impute missing data and all statistical analyses will be conducted with the available data. We also plan to include sub-group analyses for the individual subspecialties.

Any protocol amendments were first submitted to the Institutional Review Boards. Upon their approval, the new protocols were disseminated to all research staff (including all investigators, research coordinators, etc.) via email with a summary of all changes. If the amendment required additional training for study procedures, training was conducted with the applicable study staff. If the amendment changed anything about what is reported on clinicaltrials.gov, then the study’s registration on clinicaltrials.gov was updated accordingly.

In the event that a subject was harmed as a result of their participation in this study, the clinical team provided or arranged for treatment as necessary. This treatment, as well as other medical expenses, were billed to the subject or the subject’s insurance company in the usual manner. Subjects did not waive any legal rights by signing the informed consent form for this study.

### Dissemination

Once the study has entered the data reporting phase, the datasets used and/or analysed during the current study will be available from the corresponding author on reasonable request. The protocol and results will be released onto clinicaltrials.gov as is required upon study completion. Final study results will be published in a peer-reviewed, PubMed-indexed journal to reach healthcare professionals. The authors will also seek to present the study findings at relevant orthopaedic subspecialty society meetings. A website exists on the OrthoCarolina Research Institute website describing this project. Final findings and results will be published on this website for the general public, as well as disseminated through the OCRI social networking channels.

## Discussion

This study is, to our knowledge, the first to evaluate the safety and efficacy of utilizing a completely opioid-free perioperative analgesia protocol. Studies to date have combined opioid and non-opioid medications or pharmacologic alternatives in the same protocol. This study will be the first to try and distinguish the analgesic effects of opioid-including and opioid-free pain regimens. A particularly novel element of this protocol is the elimination of opioids during the surgical procedure. It is unknown what effect opioid administration during anesthesia has on patients with regards to known opioid-related side effects including post-operative delirium, nausea, and constipation. An early case-series study offers encouraging results. Leas et al. (2019) [36] reported that patients undergoing elective shoulder arthroplasty and treated with non-opioid multimodal analgesia experienced overall low levels of pain at 24 h after surgery (2.5 out of 10), which remained stable at all postoperative time points. Additionally, there were low rates of reported nausea, constipation, and falls.

Another strength of the present study is the randomized study design, which limits error and bias in the results. Conclusions gained from the RCT are also potentially bolstered by the addition of the prospective observational arm. Finally, the results from this study are generalizable, as we enrolled patients from multiple orthopedic subspecialties, allowing us to draw conclusions that may impact orthopedic surgery as a whole.

The major limitation of this study is the inclusion of a select few orthopedic procedures. All surgeries were relatively straightforward (i.e. no revision surgeries) and commonly performed procedures. Patients who are optimal candidates for these elective surgeries may be healthier, less likely to experience post-operative complications, and/or more likely to be opioid-naïve than other orthopedic populations (e.g. trauma or oncology patients). Complicated procedures may also inherently be experienced as more painful. The orthopedic community will need to establish the efficacy of opioid-free multi-modal analgesic protocols in a broader range of patients and procedures, including complex operations and patients with significant medical comorbidities.

We recognize that there are some substantial barriers to establishing completely opioid-free analgesic pathways in practice. When pain was introduced as the fifth vital sign in the 1990s, a cascade of events commenced that resulted in unintended consequences of overzealous treatment of pain. Clinicians started relying on opioid medication to eliminate patients’ pain, and public misconceptions about the efficacy of opioids for treating pain grew [52, 53]. Partially, the underlying problem was a mounting lack of understanding about the risks and benefits of opioid medications. Our study attempts to address these roadblocks by providing both patients and clinicians with educational sessions explaining the relative risks and benefits of opioids, while offering alternative analgesic modalities. Increasing education to change pain-management culture will ultimately need to be carried out on a large scale to create population-level change.

Finally, our immediate future plan is to complete enrollment for this present study. Once this single-institution investigation is complete, we will analyze our results. Provided this study demonstrates safety and efficacy, we will launch a population health study with protocol implementation across a large, state-wide healthcare system.

## Summary and conclusions

We describe the detailed protocols used in the first multi-specialty orthopedic RCT comparing totally opioid-free perioperative pain management to traditional analgesic pathways. This represents an initial step towards demonstrating that non-opioid multimodal protocols can provide a safe and predictable pathway for patients undergoing elective surgery. Our goal in reporting these pathways is so that they may be implemented at other institutions and utilized in the nationwide fight against the opioid epidemic.

## Data Availability

The study corresponding to this manuscript is still in the recruitment phase. Therefore, no available data currently exists to be shared. However, once the study has entered the data reporting phase, the datasets used and/or analysed during the current study will be available from the corresponding author on reasonable request. The protocol and results will be released onto clinicaltrials.gov as is required upon study completion. There are no contractual agreements limiting investigator access to study data.

## Abbreviations

ACDA: Anterior cervical disc arthroplasty
ACDF: Anterior cervical discectomy and fusion
CMC: Carpometacarpal
CrCl: Creatinine clearance
NNT: Number needed to treat
NRS: Numeric rating scale
NSAIDs: Non-steroidal anti-inflammatories
RCT: Randomized controlled trial
RTSA: Reverse total shoulder arthroplasty
THA: Total hip arthroplasty
TSA: Total shoulder arthroplasty
